# Supplementary material for: Tumor–Stromal Interactions in a Co-Culture Model of Human Pancreatic Adenocarcinoma Cells and Fibroblasts and Their Connection with Tumor Spread
Source: Biomedicines. 2021 Mar 31;9(4):364. doi: 10.3390/biomedicines9040364 (PMC8065458; doi:10.3390/biomedicines9040364)
Supplement: Supplementary file 1 [file biomedicines-09-00364-s001.pdf]

Figure S1 Cytokines Array map (**A**). Images of the chemiluminescence signals on membranes (**B**)

A

Each antibody is spotted in duplicate vertically

	A	B	C	D	E	F	G	H
1, 2	POS	POS	NEG	NEG	GCSF	GM-CSF	GRO a/b/g	GRO alpha (CXCL1)
3, 4	IL-1 alpha (IL-1 F1)	IL-2	IL-3	IL-5	IL-6	IL-7	IL-8 (CXCL8)	IL-10
5, 6	IL-13	IL-15	IFN-gamma	MCP-1 (CCL2)	MCP-2 (CCL8)	MCP-3 (CCL7)	MIG (CXCL9)	RANTES (CCL5)
7, 8	TGF beta 1	TNF alpha	TNF beta	BLANK	BLANK	BLANK	BLANK	POS

B

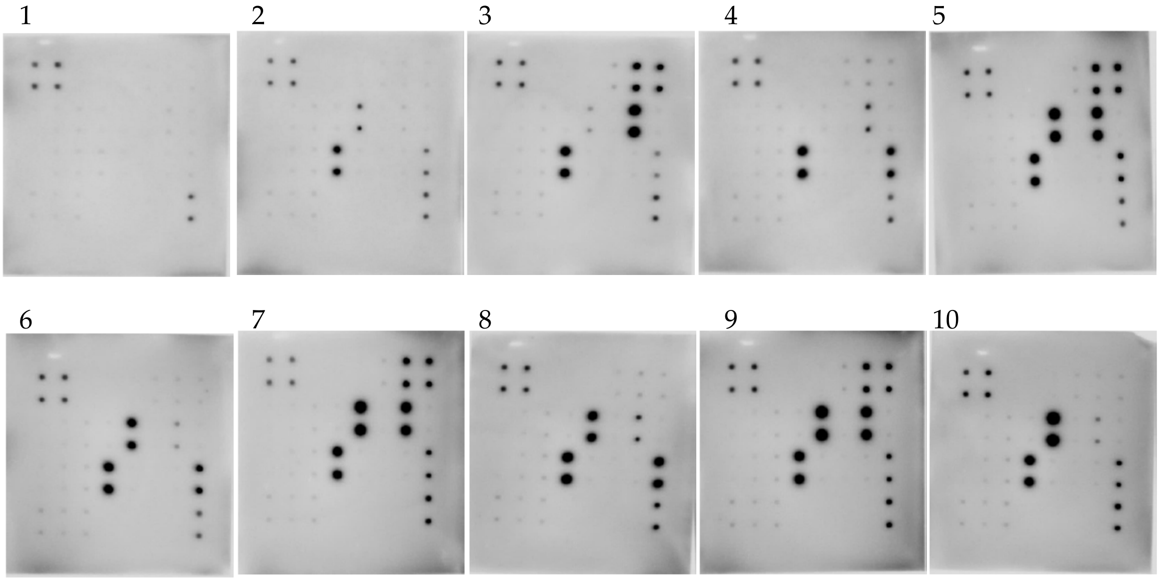

1. Uncultured medium sample as negative control.
2. Supernatant of LC5 cells monoculture.
3. Supernatant of Capan-1 monoculture.
4. Supernatant of PL-45 monoculture.
5. Supernatant of Capan-1 and LC5 direct co-culture.
6. Supernatant of PL45 and LC5 direct co-culture.
7. Supernatant of LC5 cells cultured with Capan-1 conditioned medium.
8. Supernatant of LC5 cells cultured with PL-45 conditioned medium.
9. Supernatant of Capan-1 cells cultured with LC5 conditioned medium.
10. Supernatant of PL-45 cells cultured with LC5 conditioned medium.

Table S1. Differential characteristics between tumor cell lines included in the study

	**Capan-1**	**PL-45**	**References**
Tissue	Pancreas; derived from metastatic site (liver)	Pancreatic adenocarcinoma (primary tumor)	https://www.lgcstandards-atcc.org/products/all/HTB-79.aspx?geo_country=es# https://www.lgcstandards-atcc.org/products/all/CRL-2558.aspx?geo_country=es#characteristics
K-RAS	Mutation	Mutation	Berrozpe et al. 1994<br>Jaffee et al. 1998<br>Kita et al. 1999<br>Butz et al. 2003<br>Li et al. 2010
CDKN2A/p16	Homozygous deletion	Promoter methylation	Caldas et al. 1994<br>Huang et al. 1996<br>Li et al. 2010
TP53	Mutation	Mutation	Berrozpe et al. 1994<br>Huang et al. 1996<br>Li et al. 2010
SMAD4/DPC4	Null	Wild type	Schutte et al. 1996<br>Su et al. 2001<br>Deer et al. 2010<br>Li et al. 2010<br>Dempe et al. 2010

Berrozpe G, Schaeffer J, Peinado MA, Real FX, Perucho M. Comparative analysis of mutations in the p53 and K-ras genes in pancreatic cancer. Int J Cancer. 1994; 58(2):185-91.

Butz J. Wickstrom E, Edwards J. Characterization of mutations and loss of heterozygosity of p53 and K-ras2 in pancreatic cancer cell lines by immobilized polymerase chain reaction. BMC Biotechnology. 2003; .3-11.

Caldas C, Hahn SA, da Costa LT, Redston MS, Schutte M, Seymour AB, et al. Frequent somatic mutations and homozygous deletions of the p16 (MTS1) gene in pancreatic adenocarcinoma. Nat Genet. 1994; 8(1):27-32.

Deer EL, Gonzalez-Hernandez J. Coursen JD, Shea JE, Ngatia J, Scaife CL, et al. Phenotype and genotype of pancreatic cancer cell lines. Pancreas. 2010; 39(4):425-35.

Dempe S. Stroh-Dege AY, Schwarz E, Rommelaere J, Dinsart C. SMAD4: a predictive marker of PDAC cell permissiveness for oncolytic infection with parvovirus H-1PV. Int J Cancer. 2010; 126(12):2914-27.

Huang L, Goodrow TL, Zhang SY, Kein-Szanto AJ, Chang H, Ruggeri BA. Deletion and mutation analyses of the P16/MTS-1 tumor suppressor gene in human ductal pancreatic cancer reveals a higher frequency of abnormalities in tumor-derived cell lines than in primary ductal adenocarcinomas. Cancer Res. 1996; 56(1):1137-41.

Jaffee EM, Schutte M, Gossett J, Morsberger LA, Adler AJ, Thomas M, et al. Development and characterization of a cytokine-secreting pancreatic adenocarcinoma vaccine from primary tumors for use in clinical trials. Cancer J Sci Am. 1998; 4(3):194-203.

Kita K, Saito S, Morioka CY, Watanabe A. Growth inhibition of human pancreatic cancer cell lines by anti-sense oligonucleotides specific to mutates K-ras genes. Int J Cancer. 1999; 80(4):553-8.

Li J. Wientjes MG, Au JL. Pancreatic cancer: pathobiology, treatment options, and drug delivery. AAPS J. 2010; 12(2):223-32.

Schutte M. Hruban RH, Hedrick L, Cho KR, Nadasdy GM, Weinstein CL, et al. DPC4 gene in various tumor types. Cancer Res. 1996; 56(11):2527-30.

Su GH, Gansal R, Murphy KM, Montgomery E, Yeo CJ, Hruban RH, et al. ACVR1B (ALK4, activin receptor type 1B) gene mutations in pancreatic carcinoma. Proc Natl Acad Sci USA. 2001; 98(6):3254-7.
